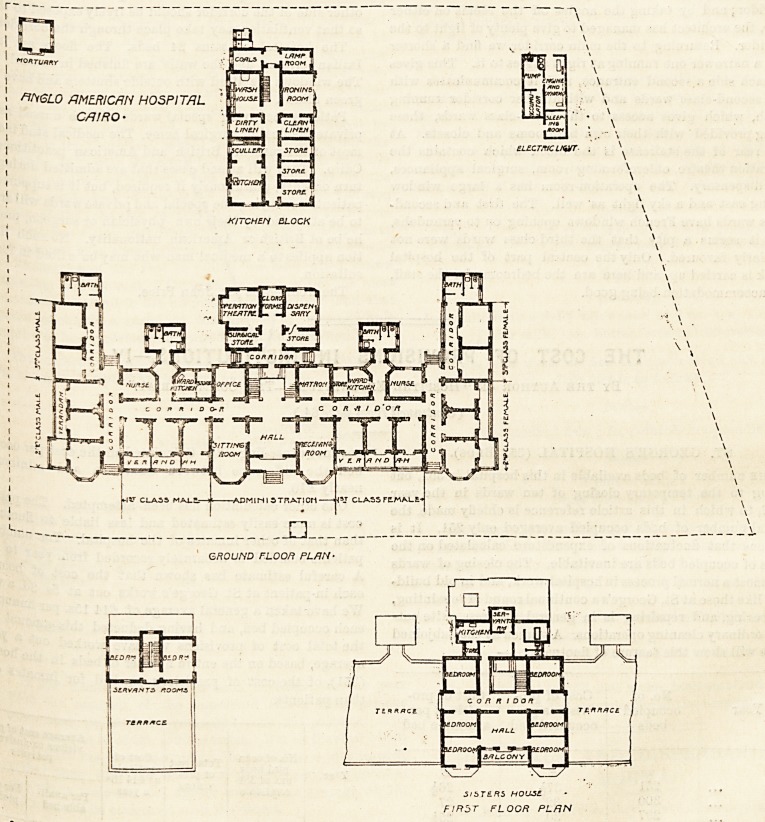# Anglo-American Hospital in Cairo

**Published:** 1904-04-30

**Authors:** 


### HOSPITAL ADMINISTRATION.

### CONSTRUCTION AND ECONOMICS.

### ANGLO-AMERICAN HOSPITAL IN CAIRO.

This hospital was lately opened by Lady Cromer. Subscriptions towards the cost of the building were made or collected by the principal English and American residents; but Sir Ernest Cassel met every subscription and donation with the gift of a similar sum. The institution is under the patronage of the King and Queen of England and the President of the United States, while Lord Cromer is the chairman of the general committee and the Consul-General of the United States is vice-chairman.

Although preference is given to English and American patients, there is no restriction as to the admission of natives of other countries. It is intended that the hospital shall be self-supporting; and it is planned to accommodate persons in different circumstances. To this end the wards have been classed as special, private, and general, and the charges vary from one guinea a week to one guinea a day. There is, however, a clause in the regulations whereby patients without means may be admitted on special terms if sent in by the British or American Consuls. There is also under consideration an arrangement with the military authorities by which the wives and children of English soldiers quartered in Cairo may be admitted. The aims which the hospital has in view are therefore of a very wide range, and make us doubt whether it is large enough to bear the strain which is sure to be placed upon it sooner or later.

The building consists of three blocks: the hospital proper, the kitchen and laundry department, and the electric light installation. There is also a detached mortuary.

The hospital proper is in plan like the letter E; and the left side of the letter faces north and contains the hall, the receiving-room, the sitting-room, and on each hand are three small wards for first-class patients—those on the right being for women, and on the left for men. South of these above-named rooms a corridor runs, and behind this are the staircase, the matron's room, the office, store, ward, kitchen and nurses' room, all these being in duplicate except the staircase. Between the ward kitchen and the nurses' room there is a short corridor from which project the bathroom and closets, which are well cut off by a cross-ventilated corridor; and by taking the angles off the rooms on either side, the architect has managed to give plenty of light to the corridor. Returning to the main corridor we find a shorter and a narrower one running at right angles to it. This gives on each side a second entrance, and it communicates with the second-class wards and with another corridor running south, which gives access to the third-class wards, these being provided with their own bathrooms and closets. At the rear of the staircase is the block which contains the operation theatre, chloroforming room, surgical appliances, and dispensary. The operation-room has a large window facing east and a sky light as well. The first and second-class wards have French windows opening on to verandahs, and it seems a pity that the third-class wards were not similarly favoured. Only the central part of the hospital block is carried up, and here are the bedrooms for the staff, the accommodation being good.

The critic who examines this plan will be struck with its compactness, but he will probably ask, why was the building so much compressed when it is surrounded by a large open space, and when the climate of Egypt has to be considered? One ward on each side, for second-class patients, has a bay window to the north and another window to the east (or west); but the other wards of this section and all the first-class wards have no cross-ventilation except what may be obtained from the corridor if the ward doors be kept open or if fanlights be provided; and even then it cannot be much, as the corridor itself is blocked on both sides. The third-class wards appear to be the best ventilated of the lot. It may be laid down as certain that when the contingencies of hospital construction require the presence of a corridor on one side of the wards (as was most likely the case in Cairo), the other side of the corridor should be freely exposed to the air, so that ventilation may take place through the corridor.

The hospital contains 24 beds. The floors are laid in Italian terrazzo, and the walls are finished in enamel paint. The windows are fitted with outside shutters and have dark-green blinds.

Patients occupying special wards will be nursed as in a private medical or surgical home. The medical staff includes most of the resident British and American practitioners in Cairo. They will attend cases that are admitted during their turn on duty, gratuitously if required, but it is supposed that patients occupying the special and private wards will arrange to be attended by their own physician or surgeon, provided he be of British or American nationality. No such limitation applies to a medical man who may be called in for consultation.

The architect is Mr. John Price.

## Figures and Tables

**Figure f1:**